# Blimp-1 Upregulation by Multiple Ligands *via* EGFR Transactivation Inhibits Cell Migration in Keratinocytes and Squamous Cell Carcinoma

**DOI:** 10.3389/fphar.2022.763678

**Published:** 2022-02-02

**Authors:** Hyemin Lee, Duen-Yi Huang, Hua-Ching Chang, Chia-Yee Lin, Wan-Yu Ren, Yang-Shia Dai, Wan-Wan Lin

**Affiliations:** ^1^ Department of Pharmacology, College of Medicine, National Taiwan University, Taipei, Taiwan; ^2^ Department of Dermatology, Taipei Medical University Hospital, Taipei, Taiwan; ^3^ Department of Dermatology, National Taiwan University Hospital, Taipei, Taiwan; ^4^ Department and Graduate Institute of Pharmacology, National Defense Medical Center, Taipei, Taiwan; ^5^ Graduate Institute of Medical Sciences, Taipei Medical University, Taipei, Taiwan

**Keywords:** EGFR transactivation, keratinocytes, squamous cell carcinoma, migration, Blimp-1

## Abstract

B lymphocyte-induced maturation protein-1 (Blimp-1) is a transcriptional repressor and plays a crucial role in the regulation of development and functions of various immune cells. Currently, there is limited understanding about the regulation of Blimp-1 expression and cellular functions in keratinocytes and cancer cells. Previously we demonstrated that EGF can upregulate Blimp-1 gene expression in keratinocytes, playing a negative role in regulation of cell migration and inflammation. Because it remains unclear if Blimp-1 can be regulated by other stimuli beyond EGF, here we further investigated multiple stimuli for their regulation of Blimp-1 expression in keratinocytes and squamous cell carcinoma (SCC). We found that PMA, TNF-α, LPS, polyIC, H_2_O_2_ and UVB can upregulate the protein and/or mRNA levels of Blimp-1 in HaCaT and SCC cells. Concomitant EGFR activation was observed by these stimuli, and EGFR inhibitor gefitinib and Syk inhibitor can block Blimp-1 gene expression caused by PMA. Reporter assay of Blimp-1 promoter activity further indicated the involvement of AP-1 in PMA-, TNF-α-, LPS- and EGF-elicited Blimp-1 mRNA expression. Confocal microscopic data indicated the nuclear loclization of Blimp-1, and such localization was not changed by stimuli. Moreover, Blimp-1 silencing enhanced SCC cell migration. Taken together, Blimp-1 can be transcriptionally upregulated by several stimuli in keratinocytes and SCC via EGFR transactivation and AP-1 pathway. These include growth factor PMA, cytokine TNF-α, TLR ligands (LPS and polyIC), and ROS insults (H_2_O_2_ and UVB). The function of Blimp-1 as a negative regulator of cell migration in SCC can provide a new therapeutic target in SCC.

## Introduction

B lymphocyte-induced maturation protein-1 (Blimp-1) encoded by the *PRDM1* gene is a member of PRDM family. Blimp-1 is a transcriptional repressor that can regulate cell growth and differentiation. Blimp-1 lacks intrinsic histone methyltransferase activity and serves as a scaffold to epigenetically modulate DNA binding, gene silencing and chromatin reorganization (Bikoff et al., 2009; Minnich et al., 2016). Blimp-1 is a well-known master regulator required for the differentiation and function of hematopoietic lineages like B lymphocytes (Chang et al., 2000; Martins and Calame, 2008; Wang et al., 2019), T lymphocytes (Martins and Calame, 2008; Jain et al., 2016; Fu et al., 2017), dendritic cells (Chan et al., 2009), macrophages (Chang et al., 2000), and granulocytes (Chang et al., 2000). Accordingly Blimp-1 is critical in maintenance of immune homeostasis, and deficiency of Blimp-1 function may contribute to autoimmune disorders (Fu et al., 2017) and inflammation (Chiang et al., 2013).

Several studies have demonstrated the pathways to regulate Blimp-1 expression, in particular in lymphocytes. In this context, IL-21 and IL-23 which are crucial for lymphocytes differentiation and functions have been demonstrated to induce Blimp-1 expression (Ozaki et al., 2004; Jain et al., 2016). In lymphocytes, Blimp-1 expression is controlled by multiple transcriptional factors including AP-1, IRF4, STAT3, STAT5, NF-κB, FOXP3, and NFAT (Calame, 2008; Martins and Calame, 2008), and is also regulated by histone deacetylation (Tanaka et al., 2016). Study indicates that TGF-β acts as a suppressor of Blimp-1 expression during Th17 differentiation (Salehi et al., 2012). In contrast, in breast cancer cells TGF-β1 induces Blimp-1 expression via a c-Raf/ERK/AP-1 pathway and Blimp-1 mediates TGF-β-induced EMT *via* repression of BMP-5 (Romagnoli et al., 2012). On the other hands, TGF-β can induce Blimp-1 expression via Wnt/β-catenin signaling in fibroblasts (Magnusdottir et al., 2007). In addition, in macrophages and B lymphocytes Blimp-1 is a target of unfolded protein response and can be induced by PERK signaling pathway (Doody et al., 2006). Apart from transcription, Blimp-1 can be degraded by proteasome when it undergoes SUMOylation (Shimshon et al., 2011). Besides proteasomal degradation, Blimp-1 can undergo lysosomal degradation in keratinocytes (Chang et al., 2018).

Besides immune cells, Blimp-1 plays various roles in skin biology. Conditional Blimp-1 knockout in skin impedes terminal cornification (Magnusdottir et al., 2007), revealing crucial functions of Blimp-1 in skin homeostasis. Mice specifically lacking Blimp-1 in keratinocytes spontaneously develop neutrophils-predominant skin inflammation (Chiang et al., 2013). Furthermore, our study indicates that activation of EGFR can upregulate the Blimp-1 gene transcription *via* the PKC, p38, and ERK pathways in keratinocytes (Chang et al., 2018). Reciprocally the expression of Blimp-1 in keratinocytes exerts a negative role in EGF-induced inflammation and migration, and in turn controls keratinocyte differentiation via regulation of gene expression (Chang et al., 2018).

Given that Blimp-1 is involved in skin biology and can be induced by EGF and PMA in human keratinocytes (Chang et al., 2018), we were interested to further explore the regulation and function of Blimp-1 in keratinocytes and squamous cell carcinoma (SCC). Therefore, we examined several stimuli besides EGF and attempted to understand their regulation depending on EGFR activation or not. The tested stimuli included tumor promoter PMA, inflammatory response activators TNF-α and TLR ligands, and stressors H_2_O_2_ and UVB. The reasons we chose these agents are due to their functions in keratinocytes biology and skin disorders, in particular relating to EGFR activation which is the major growth factor to control keratinocyte biology (Nanba et al., 2013). PKC-dependent activation by phorbol ester PMA has been implicated in the regulation of keratinocyte differentiation and skin tumor formation, which in part depend on the EGFR activation (Snoek et al., 1987; Ando et al., 1993). TNF-α, a key cytokine in inflammatory skin disease, also has been shown to induce EGFR activation in keratinocytes (Segawa et al., 2018). TLRs, the major pattern recognition receptors for host defense, are expressed in keratinocytes (Lebre et al., 2007). On the other hands, UVB and reactive oxygen species (ROS) can lead to DNA damage-associated EGFR transactivation and inflammation in keratinocyte (Chiu et al., 2021). Moreover, the role of Blimp-1 in cell migration of keratinocytes and SCC was addressed.

## Materials and Methods

### Reagents

DMEM (high glucose; Cat. No. 12100-046) and trypsin-EDTA were from Gibco (Carlsbad, CA, United States). FBS was from HyClone (Logan, UT, United States). Penicillin-streptomycin solution and penicillin-streptomycin-amphotericin B solution were from Biological Industries (Kibbutz Beit Haemek, Israel). Poly (I:C) was from InvivoGen (San Diego, CA, United States). TNF-α was from Biolegend (San Diego, CA, United States). PMA, PBS, mitomycin C, LPS, and H_2_O_2_ were from Sigma-Aldrich (St. Louis, MO, United States). Gefitinib was from Selleckchem (Houston, TX, United States). Recombinant human EGF was from PeproTech (Rocky Hill, NJ, United States). Blimp-1 (#9115), p-EGFR (Y1068, #2234) and Syk (#2712) antibodies were from Cell Signaling (Beverly, MA, United States). EGFR antibody (sc-03) was from Santa Cruz (Santa Cruz, CA, United States). p-Syk antibody (PK1010) was from Millipore (Burlington, Ma, United States).

### Cell Culture

Human immortalized HaCaT keratinocytes, oral SCC Cal-27 and SAS cells were cultured in high glucose Dulbecco’s modified Eagle’s medium (DMEM) supplemented with 10% FBS and 1% penicillin-streptomycin-amphotericin B. NHEKs (normal human epidermal keratinocytes) were obtained from normal adult human foreskin and isolated as described previously (Chiu et al., 2021). The experiments were conducted according to the Declaration of Helsinki principles and approved by the Ethics Committee of Mackay Memorial Hospital (Institutional Review Board codes 19MMHIS173e). All cell lines were incubated at 37°C under a humidified atmosphere of 5% CO_2_ in air. HaCaT and SCC cell lines were seeded in 6-well or 12-well tissue culture plates at a density of 8 × 10^4^ cells/well.

### Generation of Knockdown Cells Using Lentiviral shRNAs

In construction of stable short hairpin RNA (shRNA) knockdown cell lines, lentiviral particles encoding shRNA targeting human *PRDM1* (Sigma-Aldrich, St. Louis, MO, United States) were used for transfection. Lentivirus-containing supernatants were harvested 24 h after transfection, filtered using a 0.45 μm filter, and diluted with fresh culture media to transduce target cells in the presence of 8 μg/ml PolyBrene (hexadimethrinebromide). Transduced cells were selected with puromycin (3 μg/ml) (Thermo Fisher Scientific, Waltham, MA, United States) for 2 weeks to select successful transfection.

### Immunoblotting

Cells were lysed by adding radioimmunoprecipitation assay (RIPA) buffer. The extracts were sonicated for 10-15 sec to complete cell lysis and shear DNA, and then centrifuged at 16,200 g, 4°C for 30 min. The protein concentrations of the supernatants were determined using the Bio-Rad protein assay. Equal amounts of the protein were loaded and electrophoresed on 8–15% SDS-PAGE, and then electro-transferred to Immobilon-P (0.45 μm PVDF; Millipore). After transfer, the membrane was blocked with Tris-buffered saline with Tween 20 (TBST) containing 5% (w/v) nonfat dry milk for 1 h at room temperature. After incubation with the primary antibodies (at the appropriate dilution as recommended in the product data sheet) with gentle agitation overnight at 4°C, the membranes were washed with TBST for three times and incubated with horseradish-peroxidase-linked secondary antibodies with gentle agitation for 1 h at room temperature. After washing with TBST for three times, the protein bands were detected on X-ray film with ECL reagents.

### Quantitative Polymerase Chain Reaction

After stimulation, cells were harvested with TriPure isolation reagent (Roche Diagnostics, Indianapolis, IN, United States) and RNA was extracted according to the manufacturer’s procedure. Total RNA (1–2 μg) was converted into cDNA by reverse transcription system kit (Promega, Heidelberg, Germany). Q-PCR was performed using FastStart SYBR Green Master (Roche Diagnostics, Indianapolis, IN, United States) in 96-well plates, and determined using ABI Prism 7900 (Applied Biosystems, Oakland, CA, United States). The primers used for human *RPDM1* were 5′-CGA​AAT​GCC​CTT​CTA​CCC​T-3′ and 5′-GCG​TTC​AAG​TAA​GCG​TAG​GA-3′ and the primers used for human *β-actin* were 5′-AGG​AAG​GCT​GGA​AGA​GTG​C-3′ and 5′-CGG​GGA​CCT​GAC​TGA​CTA​CC-3’.

### Wound Healing Assay

Cells were seeded (4 × 10^4^ cm^2^) into 12-well culture-insert purchased from ibidi (Martinsried, Germany). After attachment, the culture-insert was gently removed from each well, and the well was washed three times with PBS to remove the suspended cells. Before adding the drugs, the cells were incubated with anti-proliferative agent mitomycin C (5 μg/ml) for 30 min. Finally, cells were incubated in medium in the absence or presence of PMA (30 nM) or TNF-α (10 ng/ml) in HaCaT cells, and TNF-α (10 ng/ml) or EGF (50 ng/ml) in SCC. The process was recorded by photographs, and cell migration was quantified.

### Luciferase Assay

Blimp-(wt) and Blimp-(AP1 mt) report constructs (human Blimp-1 promoter in pGL3-basic) were gifts from Alexander Dent (Addgene plasmid # 40340 and #40341). Following the commercial standard protocol, HaCaT cells were transfected with Blimp-1 reporter plasmid and β-galactosidase expression vector by using Lipofectamine 2000 reagent (Invitrogen) and then EGF (50 ng/ml) was treated for 24 h. After harvest, the luciferase activity was determined by luciferase assay system kit (Promega, Heidelberg, Germany), followed by microplate luminometer. Luciferase activity was normalized with activity of β-galactosidase, and expressed as fold of control without stimulus treatment.

### Confocal Microscopy

HaCaT, Cal-27, and SAS cells in full serum DMEM medium were fixed with 4% paraformaldehyde and permeabilized with 0.2% Triton X-100 in PBS for 20 min. After this process, the samples were blocked with 4% BSA for 1 h and incubated with primary antibody for 2 h at room temperature or overnight at 4°C after aspiration of blocking solution. The primary antibody was then discarded and cells were washed three times with PBS. The samples were incubated with fluorochrome-conjugated secondary antibody for 1 h in the dark afterwards. Following immunostaining process, the coverslip was counterstained with 4′6-diamidino-2-phenylindole (DAPI), and mounted on microscope slides in dark. Samples were analyzed by LSM 880 confocal microscope (Zeiss).

### Statistical Analysis

Values were expressed as the mean ± S.E.M. of at least three independent experiments. Student’s t-test or one-way ANOVA was performed to analyze the statistical significance of the differences, and the P value <0.05 was considered statistically significant.

## Results

### PMA, TNF-α, LPS, UVB, and H_2_O_2_ Upregulate Blimp-1 Expression and Activate EGFR in HaCaT Keratinocytes

**FIGURE 1:**
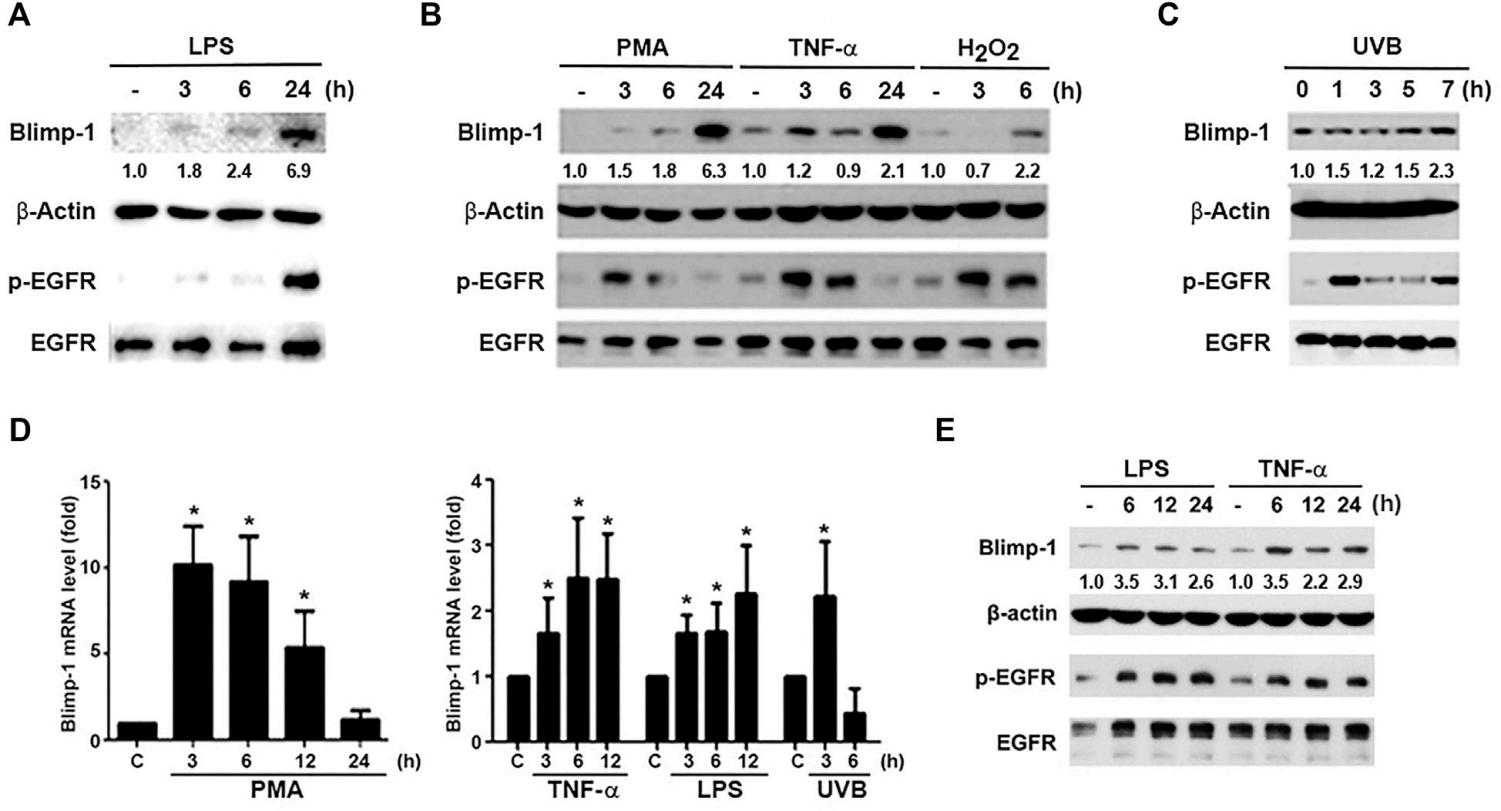
LPS, PMA, TNF-α, UVB, and H_2_O_2_ upregulate Blimp-1 gene and protein expressions in HaCaT cells and NHEKs. HaCaT cells were stimulated with LPS (1 μg/ml) **(A)**, PMA (30 nM), TNF-α (10 ng/ml), H_2_O_2_ (200 μM) **(B)** or UVB (50 mJ/cm^2^) **(C)** for indicated time periods. **(E)** NHEKs were stimulated with LPS (1 μg/ml) and TNF-α (10 ng/ml) for indicated times. After stimulation cell lysates were collected to determine Blimp-1 and β-actin expression by immunoblotting. **(D)** HaCaT cells were stimulated with PMA (30 nM), TNF-α (10 ng/ml), LPS (1 μg/ml), or UVB at 50 mJ/cm^2^ for indicated time periods and then Q-PCR was performed to evaluate the mRNA level of Blimp-1. *p < 0.05 (mean ± S.E.M., *n* = 5) as compared to control group.

To understand the regulation of Blimp-1 expression in keratinocytes, we tested several agents including tumor promoter PMA, TLR4 ligand lipopolysaccharide (LPS), cytokine TNF-α, ROS stressors H_2_O_2_ and UVB in human HaCaT keratinocytes. We found that LPS (1 μg/ml) (Figure 1A), PMA (30 nM), TNF-α (10 ng/ml) and H_2_O_2_ (200 μM) (Figure 1B) can increase Blimp-1 protein expression. Compared to other stimuli, the effect of H_2_O_2_ is much weaker. Nevertheless, polyIC (TLR3 ligand) (50 μg/ml) had no significant effect Blimp-1 expression in HaCaT cells (data not shown). On the other hands, UVB (50 mJ/cm^2^) could also induce Blimp-1 expression within 5–7 h (Figure 1C). Consistently the data from the Q-PCR study indicated that PMA, TNF-α, LPS and UVB can increase Blimp-1 mRNA level (Figure 1D).

Because previously EGF was shown to upregulate Blimp-1 expression in keratinocytes (Chang et al., 2018) and EGFR can be transactivated via extracellular and intracellular manners in various cell types including keratinocytes (Nanba et al., 2013; Wu et al., 2016), we interested to explore if Blimp-1 inducers mentioned above might exert actions related to EGFR. To this end, we determined the effects of these stimuli on EGFR expression and activation. We found that LPS (Figure 1A), PMA, TNF-α, H_2_O_2_ (Figure 1B) and UVB (Figure 1C) could activate EGFR in HaCaT cells. Strengthening these observations not only in HaCaT cells, we found that LPS and TNF-α also can induce Blimp-1 expression in NHEKs (Figure 1E). In our experimental conditions and time intervals, we ruled out the death effect of all tested agents including H_2_O_2_ and UVB in keratinocytes and SCC.

### PMA, TNF-α, TLRs Ligands, UVB, and H_2_O_2_ Upregulate Blimp-1 Expression and Activate EGFR in Cal-27 and SAS Cells

**FIGURE 2:**
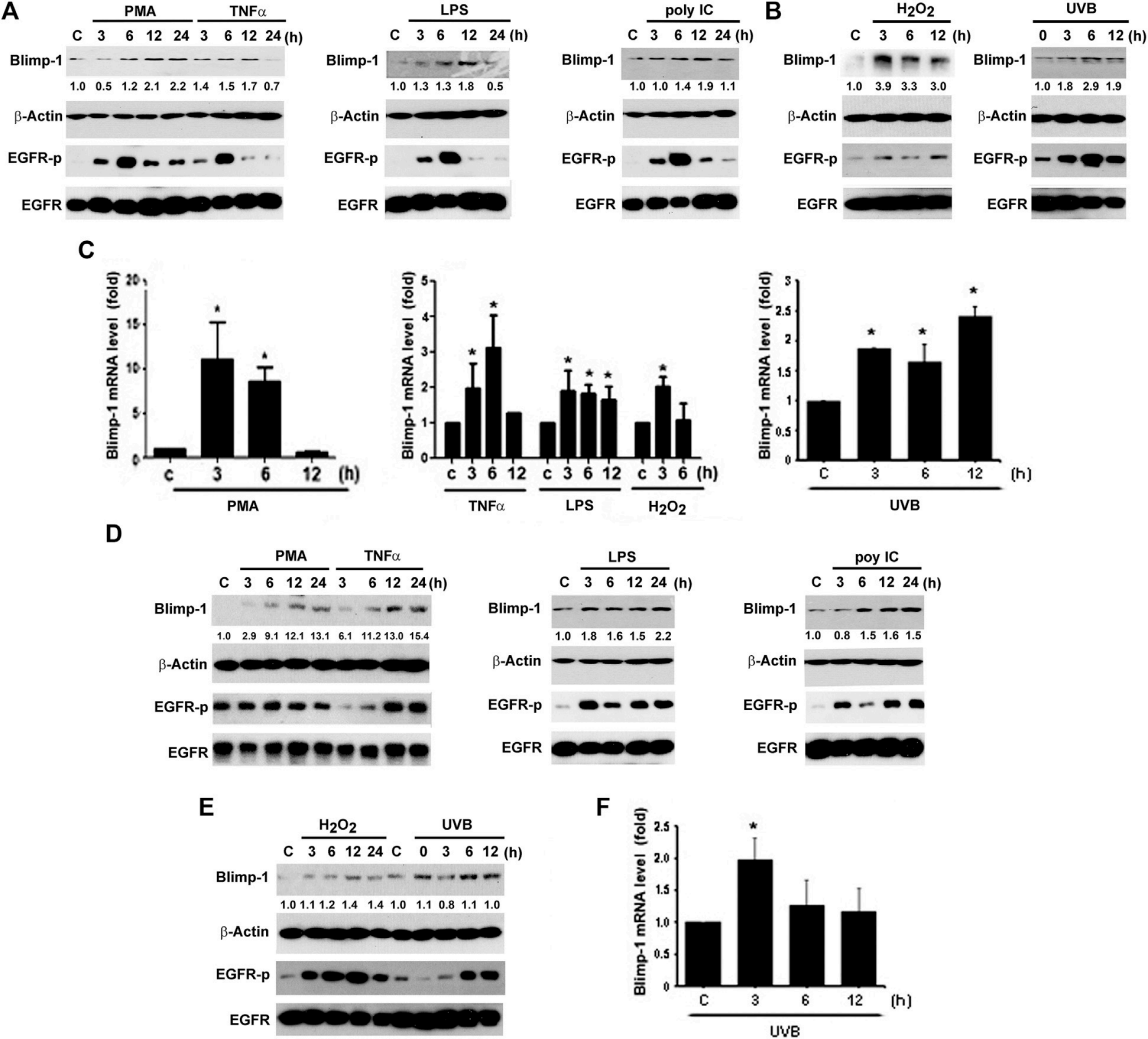
Blimp-1 expression was induced by various stimuli in Cal-27 and SAS cells. Cal-27 cells **(A–C)** and SAS cells **(D–F)** were treated with PMA (30 nM), TNF-α (10 ng/ml), LPS (1 μg/ml), polyIC (50 μg/ml), H_2_O_2_ (200 μM) or UVB (50 mJ/cm^2^) for indicated times. Protein expression of Blimp-1 and β-actin were analyzed by immunoblotting **(A,B,D,E)**. Q-PCR was performed to evaluate the mRNA level of Blimp-1 **(C,F)**. *p < 0.05 (mean ± S.E.M., *n* = 3) as compared to control group.

Besides keratinocytes, we chose two SCC cell lines Cal-27 and SAS to understand the Blimp-1 regulation. In the same manners as seen in HaCaT cells, PMA, TNF-α, LPS, H_2_O_2_ and UVB could time-dependently increase Blimp-1 protein expression in Cal-27 cells (Figures 2A,B). Unlike HaCaT cells where polyIC failed to increase Blimp-1 protein, we found it can exert this action in Cal-27 cells (Figure 2A). In Q-PCR study, our data revealed the abilities of PMA, LPS, TNF-α, H_2_O_2_ and UVB to upregulate Blimp-1 gene expression (Figure 2C). Moreover, in SAS cells PMA, TNF-α, LPS, polyIC, H_2_O_2_ and UVB all increased Blimp-1 protein expression (Figures 2D,E), and UVB also increased Blimp-1 mRNA level (Figure 2F). All these data suggest that Blimp-1 can be induced by PMA, TNF-α, LPS, H_2_O_2_ and UVB in both keratinocytes and SCC. Similarly, we also determined EGFR activation in SCC cells. We found that PMA, TNF-α, LPS, polyIC, H_2_O_2_ and UVB all activated EGFR in Cal-27 cells (Figures 2A,B). In SAS cells, the EGFR was also activated by PMA, TNF-α, LPS, polyIC, H_2_O_2_ and UVB (Figures 2D,E). All these findings indicate that transactivation of EGFR is induced by all Blimp-1 regulators, suggesting the role of EGFR activation in Blimp-1 gene expression as we previously reported in the case of exogenous EGF (Chang et al., 2018).

### EGFR Activation Mediates Blimp-1 Gene Expression *via* AP-1 Activation

**FIGURE 3:**
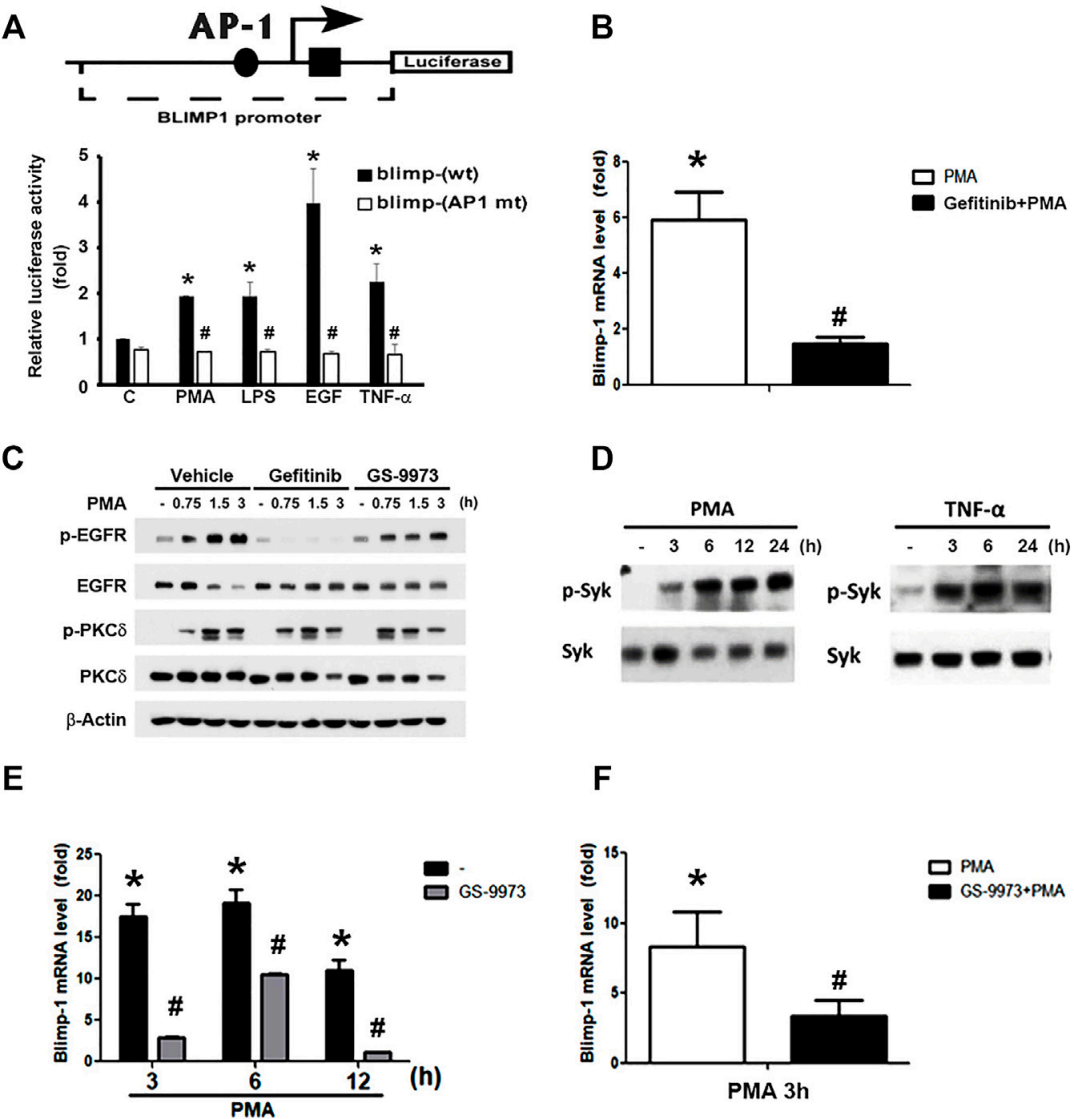
Roles of EGFR and Syk activation in PMA- and TNF-α-induced Blimp-1 gene expression. **(A)** The indicated luciferase constructs in lentiviral vectors were transiently transfected in HaCaT cells and then PMA (30 nM), LPS (1 μg/ml), EGF (50 ng/ml), and TNF-α (10 ng/ml) was treated for 15 h. After harvesting, the luciferase luminescence was measured. Bars showed means ± S.E.M. (*n* = 3). *p < 0.05 as compared to control group. #*p* < 0.05, indicating the abolishment of agent-induced luciferase activity by AP-1 deletion. **(B)** After pretreatment with gefitinib (1 uM) for 30 min, Cal-27 cells were treated with PMA (30 nM) for 3 h, and then mRNA level of Blimp-1 was evaluated by Q-PCR. **(C)** HaCaT cells were pre-treated with gefitinib (1 uM) or GS-9973 (1 uM) for 30 min prior to the stimulation with PMA (30 nM). HaCaT cells **(D,E)** and Cal-27 cells **(F)** were stimulated with PMA (30 nM) or TNF-α (10 ng/ml) for indicated time periods, and in some experiments GS-9973 (1 μM) was pre-treated for 30 min **(E,F)**. EGFR, PKCδ and Syk were analyzed by immunoblotting **(C,D)**, and Q-PCR was performed to evaluate the mRNA levels of Blimp-1 **(E,F)**. *p < 0.05 (mean ± S.E.M., *n* = 3), as compared to control group. #*p* < 0.05, indicating the significant inhibitory effects of AP-1 mutation, gefitinib and GS-9973 on Blimp-1 gene transcription.

Previous studies indicated that AP-1 is involved in BCR-mediated and TGF-β-induced Blimp-1 gene expression in B lymphocytes (Calame, 2008) and breast cancer cells (Romagnoli et al., 2012), respectively. We also found that PMA-induced Blimp-1 is dependent on EGFR transactivation in keratinocytes, because EGFR inhibitor gefitinib can reduce this effect (Chang et al., 2018). Here we used reporter assay to check if AP-1 is required for Blimp-1 expression caused by various agents in keratinocytes. As shown in Figure 3A, PMA, LPS, TNF-α and EGF treatment could increase the luciferase activity of Blimp-1 in HaCaT cells with the highest effect of EGF, and AP-1 mutation abolished these effects of different agents (Figure 3A). In addition, we also observed the ability of gefitinib to reduce PMA-induced Blimp-1 gene expression in Cal-27 cells (Figure 3B). To clarify if gefitinib might have off-target effect on PMA signaling, we used PKCδ phosphorylation as the index of PKC activation. We found that gefitinib did not change PMA-induced PKCδ phosphorylation in Cal-27 cells (Figure 3C).

Moreover, previously we found that Syk is not only an upstream signaling molecule of EGFR in keratinocytes (Wu et al., 2016) but also can be activated by PKC in monocytes (Chang et al., 2012). Therefore, we wonder if Syk is involved in the action of PMA for Blimp-1 expression. First, as our previous study showing the effect of PMA on Syk activation in monocytes (Chang et al., 2012), we found that in HaCaT cells, PMA could also increase active phospho-Syk level (Figure 3D, left panel) and Syk inhibitor GS-9973 could block PMA-induced Blimp-1 upregulation (Figure 3E). Likewise, TNF-α also can activate Syk (Figure 3D, right panel). In Cal-27 cells, GS-9973 was also found to inhibit PMA-induced Blimp-1 gene expression (Figure 3F) as well as EGFR activation (Figure 3C). These findings suggest the involvement of PKC-Syk axis in PMA-induced EGFR transactivation and subsequent Blimp-1 gene expression.

### Nuclear Localization of Blimp-1 in Keratinocytes and SAS Cells

**FIGURE 4:**
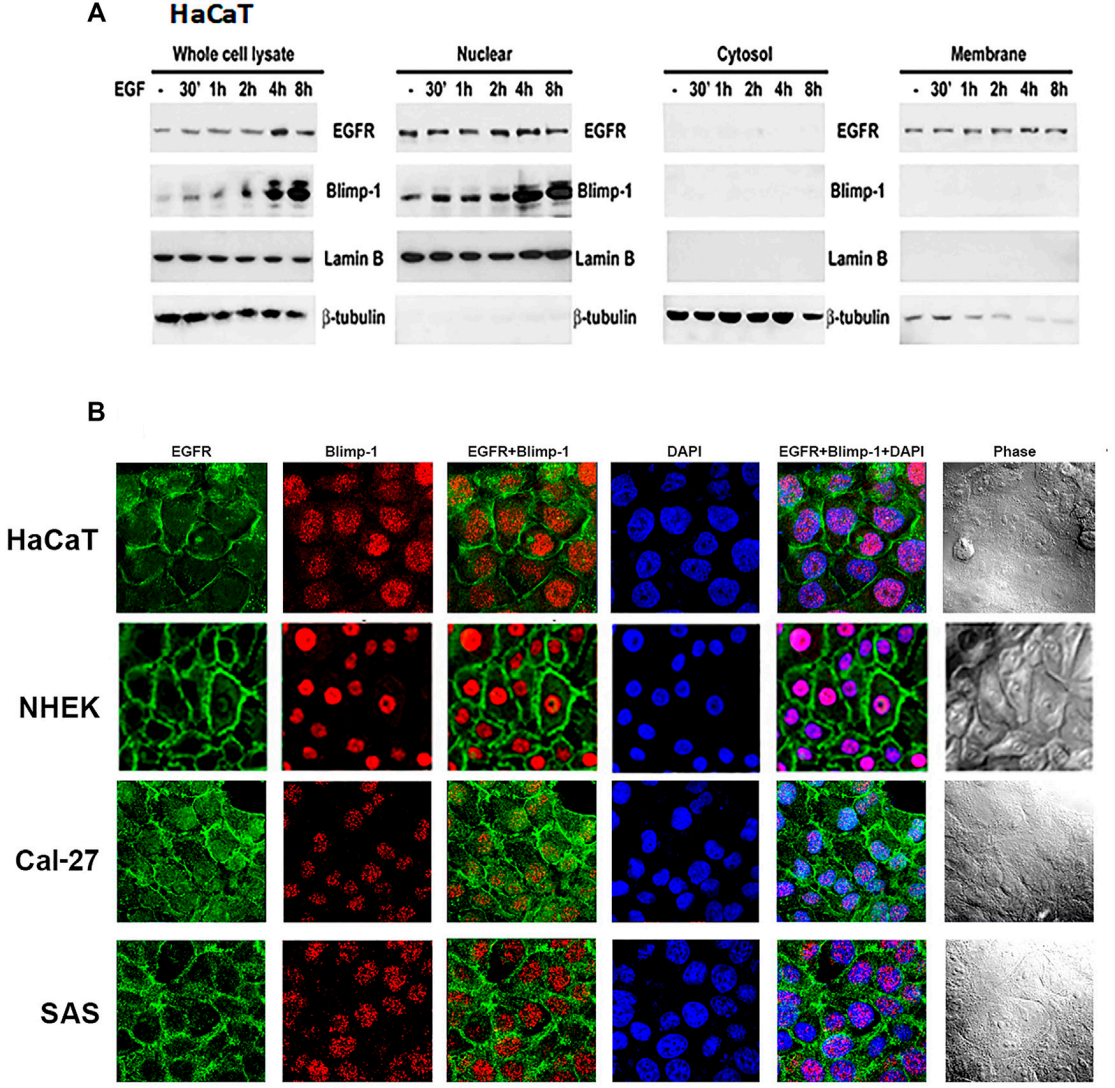
Intracellular localization of Blimp-1 in keratinocytes and SCC. **(A)** Total cell lysates of EGF-stimulated HaCaT cells were separated into cytosol, membrane and nuclear fractions. Protein levels of Blimp-1, EGFR, lamin B and β-tubulin were determined. Lamin B and β-tubulin were the nuclear and cytosol markers, respectively. Data were representative from three independent experiments. **(B)** HaCaT cells, NHEKs, Cal-27, and SAS cells were seeded in 12-well plates. Immunofluorescence staining was performed to detect EGFR and Blimp-1 expression. Nuclei were counterstained with DAPI (blue color).

After observing the relationship between Blimp-1 expression and EGFR activity, we determined the subcellular localization of Blimp-1 and EGFR in keratinocytes and SCC. Data of immunoblotting analysis of subcellular fractions revealed that Blimp-1 is located in the nuclei and EGFR is present in both nuclei and plasma membrane in HaCaT cells (Figure 4A). Data of confocal microscopy revealed that Blimp-1 and EGFR are mainly present in the nuclei and plasma membrane, respectively (Figure 4B). Similar intracellular distributions of Blimp-1 and EGFR were also observed in NHEK. In addition, PMA and TNF-α treatment for 6 h cannot alter both proteins’ localization in HaCaT cells (data not shown). Besides we checked the subcellular location of Blimp-1 and EGFR in Cal-27 and SAS cells before and after stimuli treatment. We found that Blimp-1 is localized in the nuclei of both cancer cell lines (Figure 4B) and is still kept in the nuclei after PMA, TNF-α or UVB stimulation (Supplementary Figure S1).

### Blimp-1 Negatively Regulates Cell Migration in SCC Cells But Not HaCaT Cells

**FIGURE 5:**
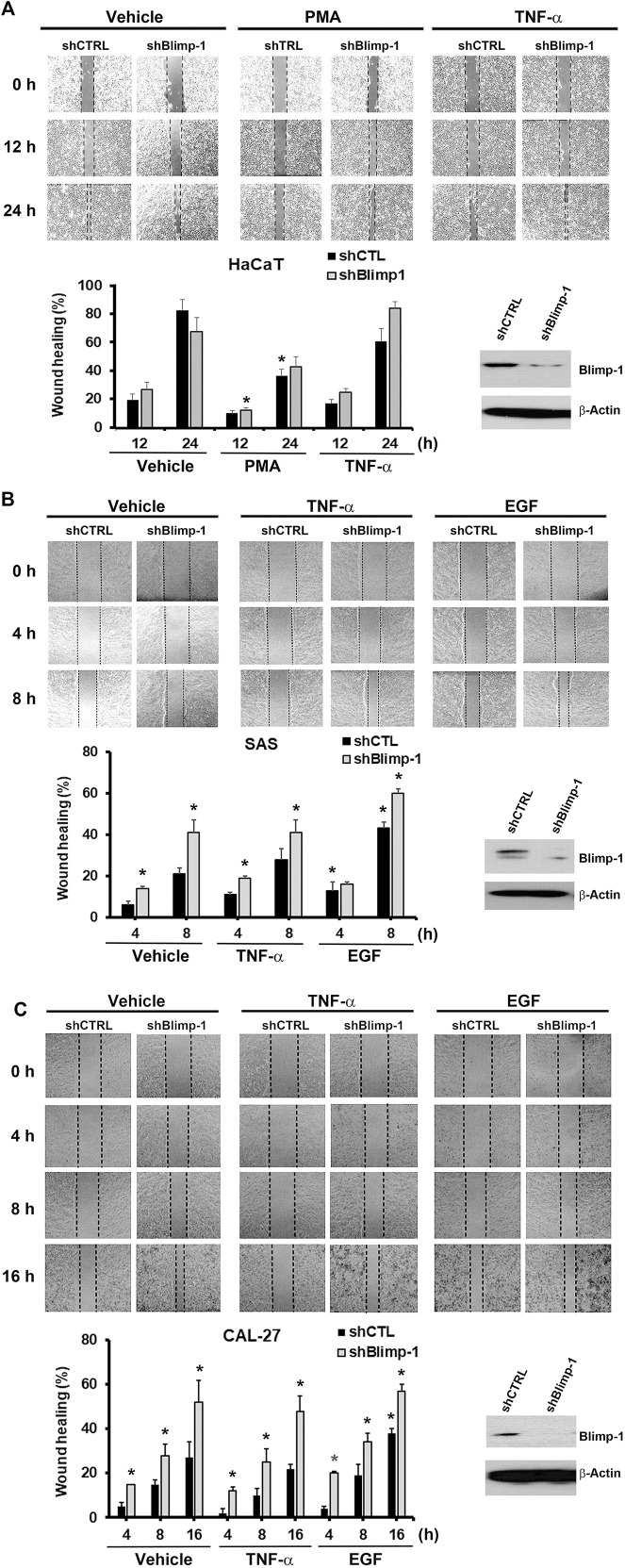
Blimp-1 negatively regulates cell migration in SCC but not HaCaT cells. After transfection of silencing Blimp-1 and control shRNA, HaCaT **(A)**, SAS **(B)** and Cal-27 **(C)** cells were seeded in wound-healing assay kit and grown overnight for attachment. Then the kits were removed and fresh DMEM medium with mitomycin C (5 μg/ml) was treated. After 30 min, cells were stimulated with PMA (30 nM), TNF-α (10 ng/ml) or EGF (50 ng/ml). Photography (100x) was taken by microscopy. Dashed lines represent boundaries of the wounds. The percentage of wound closure from the denuded gap after incubation for different times was determined. *p < 0.05 (mean ± S.E.M., *n* = 3), indicating the significant enhancement effects of shBlimp-1 and EGF, and the inhibitory effect of PMA on cell migration as compared to control cells.

Next to know the role of Blimp-1 in cell migration, we knocked down Blimp-1 in HaCaT cells using shRNA. We found silencing of Blimp-1 increases wound closure percentages as compared to control group in SAS (Figure 5B) and Cal-27 cells (Figure 5C), but not in HaCaT cells (Figure 5A). PMA slightly inhibited the cell migration of HaCaT keratinocytes. The inhibitory effect of PMA is similar to previous finding and possibly is due to the keratinocyte differentiation action of PMA (Ando et al., 1993). In both SCC, EGF but not TNF-α can promote cell migration. shBlimp-1 induced a significant enhancement on cell migration in both types of SCC, and this effect was still observed upon TNF-α treatment and was non-additive to the stimulating response of EGF (Figures 5B,C). These effects indicate that Blimp-1 is a negative regulator of cell migration in SCC.

## Discussion

Blimp-1 was initially identified as a post viral induction repressor of transcription of IFNB1 (Keller and Maniatis, 1991). Later on accumulating evidence suggests Blimp-1 as an essential regulator of immune cells differentiation, particularly in B and T lymphocytes (Nutt et al., 2007). Although so far the roles of Blimp-1 in various cell types beyond immune cells remain largely unclear, some studies have demonstrated the pathways to regulate Blimp-1 gene expression. In this aspect, we previously demonstrated that EGF can increase Blimp1 gene transcription in keratinocytes through PKC-p38, ERK signaling pathway (Chang et al., 2018). Apart from EGF, Blimp-1 is also induced by TGF-β *via* Wnt/β-catenin signaling to regulate hair follicle growth (Telerman et al., 2017). Also in breast cancer cells TGF-β1 induces Blimp-1 expression via the c-Raf/Erk/AP-1 pathway (Romagnoli et al., 2012). Notably, analyses using microarray datasets in Oncomine reveal an elevated Blimp-1 mRNA expression in samples of tongue squamous cell carcinoma (Yu et al., 2012), correlating to the high frequencies of EGFR overexpression in squamous cell carcinomas (Molinolo et al., 2009). Moreover, interaction of Reishi-F3 with TLR4/TLR2 followed by signaling through p38 MAPK is involved in the induction of Blimp-1 mRNA level (Lin et al., 2006). Blimp-1 is promptly induced in plasmacytoid dendritic cells after exposure to TLR7 and TLR9 ligands *via* a unique Ras-related C3 botulinum toxin substrate (Rac)-mediated pathway (Ko et al., 2018). Blimp-1 is also greatly induced in bone marrow-derived dendritic cells cultured with LPS, TNF-α, CpG, and poly I:C (Chan et al., 2009). All these findings suggest that Blimp-1 gene induction might be highly responsive to multiple stimuli and pathophysiological conditions.

To date studies on Blimp-1 are quite few in keratinocytes and cancer cells, and only EGFR activation is known to mediate Blimp-1 expression in keratinocytes (Chiang et al., 2013; Chang et al., 2018). The factors capable of inducing Blimp-1 and action mechanisms in relation with EGFR are still insufficiently investigated in keratinocytes and cancer cells. In this study, we examined several stimuli, and found that PKC activator PMA, cytokine TNF-α, TLRs ligands (LPS and polyIC), ROS stressors H_2_O_2_ and UVB can upregulate Blimp-1 protein expression in HaCaT and SCC (Cal-27, SAS) with different extents. Our current data indicate that Blimp-1 increase caused by these factors results from the gene transcription. Some transcriptional factors for Blimp-1 gene expression like AP-1, NF-κB, and IRF4 have been identified (Calame, 2008; Martins and Calame, 2008). Our data indicate that AP-1 is indispensable for EGF-, TNF-α-, PMA- and LPS-induced Blimp-1 gene expression in keratinocytes. Of note, we further demonstrated that EGFR activation contributes to Blimp-1 gene expression caused by these stimuli in these cell types. First of all we found that most stimulation conditions that we tested also can trigger EGFR activation. This is evidenced by the increasing EGFR phosphorylation upon treatment of LPS, PMA, TNF-α, H_2_O_2_, and UVB in HaCaT, Cal-27 and/or SAS cells. Second, these treatments do not affect the protein level of EGFR but increase EGFR phosphorylation of a late onset prior to Blimp-1 induction. Third, EGFR TKI gefitinib can block Blimp-1 induction caused by PMA. The no effect of gefitinib on PMA-induced PKC activation rules out the possibly non-specific action of gefitinib beyond EGFR. Therefore, we suggest that EGFR transactivation is occurred under these treatments and orchestrates an essential signal for Blimp-1 gene expression.

The EGFR and its ligands are recognized to centrally involve in the growth and repair process of epithelia and in carcinogenesis. Constitutive EGFR activation via ligand shedding as well as ligand-independent EGFR transactivation in keratinocytes and some EGFR-dependent cancer cell types including SCC has been reported. In keratinocytes, UVB can activate EGFR signaling by inducing shedding of EGFR ligand like HB-EGF (Pastore et al., 2008), which in turn regulates oxidative stress and inflammation (Seo and Juhnn, 2010; El-Abaseri et al., 2013; Chiu et al., 2021). Vice versa, ROS production in response to UVB or arsenite can reciprocally promote EGFR transactivation (Tseng et al., 2012; Chiu et al., 2021). Additionally, proinflammatory cytokines like TNF-α can transactivate EGFR via ERK signaling and EGFR ligand shedding in keratinocytes (Ziv et al., 2008; Potapovich et al., 2011; Wu et al., 2013; Segawa et al., 2018), and IL-1β also can induce EGFR-dependent MMP-1 expression in keratinocytes (Wan et al., 2001). On the other hand, EGFR transactivation in SCC is similarly demonstrated in response to H_2_O_2_ (Finch et al., 2006), PMA (Moon et al., 1984), LPS (Shuyi et al., 2011), UV radiation (Rodust et al., 2009), and TNF-α (Donato et al., 1989). PMA was shown to induce secretion of EGFR ligand TGF-α in A431 cells (Thornley and Jones, 1992). In head and neck SCC biopsies TLR4 expression is correlated to EGFR, and the amplifying crosstalk between EGFR and TLR4 signaling pathways leads to anti-EGFR therapy resistance (Ju et al., 2020). Taken together, because of the well-known ERK-dependent EGFR activation and EGFR-ERK pathway for AP-1 activation, we suggest EGFR-ERK-AP-1 pathway contributes to Blimp-1 gene transcription in response to the stimuli tested in this study.

In this study, we also highlight the role of Syk in PMA-dependent Blimp-1 expression. We found that Syk activity is increased by PMA and TNF-α in HaCaT cells, and Syk inhibitor GS-9973 could suppress PMA-induced Blimp-1 increase in both HaCaT and Cal-27 cells. Because PMA can activate Syk in monocytes (Chang et al., 2012), and EGFR activation also transduces Syk signaling in keratinocytes (Wu et al., 2016) and SCC (Huang et al., 2020), we treated GS-9973 and took PMA as an example to understand the signaling cascade among PKC, Syk and EGFR. Our data that GS-9973 can reduce PMA-induced EGFR-p without affecting PKC activation suggest that PKC-Syk-EGFR-ERK-AP-1 signaling pathway is involved in Blimp-1 gene transcription.

According to published data, the role of Blimp-1 in cell migration remains controversial. Blimp-1 is a negative regulator of NHEKs migration (Chang et al., 2018), but promotes breast cancer cell motility and metastasis (Sciortino et al., 2017). In SAS and Cal-27 cells, we found Blimp-1 also acts as a negative regulator of cell migration, but does not affect HaCaT cell migration, and PMA as reported inhibits keratinocyte mobility possibly due to induction of keratinocyte differentiation (Ando et al., 1993). It remains unclear for the different role of Blimp-1 in keratinocyte migration between primary NHEKs and HaCaT cell line. EGF can enhance cell migration in SCC, and this action of EGF is non-additive to the effect of Blimp-1 silencing. Collectively, Blimp-1 functioning to regulate cell migration is cell type dependent. Currently it remains unclear how Blimp-1 negatively regulates cell migration, and future study on the molecular mechanisms underlying this event is required.

## Conclusion

We demonstrate that the transcriptional regulator Blimp-1 can be transcriptionally upregulated by various stimuli including PKC activator, proinflammatory cytokine, TLR ligands, ROS and UVB in keratinocytes and SCC. The common mechanism of Blimp-1 gene induction is via the EGFR transactivation, which evokes ERK-AP-1 pathway for activation of Blimp-1 promoter. We further show that Blimp-1 can negatively regulate SCC cell migration. Combining our previous findings that Blimp-1 negatively regulates inflammation and keratinocyte differentiation, Blimp-1 is suggested to be a potential target to develop new intervention in therapy of skin diseases.

## Data Availability

The raw data supporting the conclusion of this article will be made available by the authors, without undue reservation.

## Supplementary Material

Supplementary Figure S1 PMA, UVB and TNF-a do not change intracellular Blimp-1 localization in SCC cells. Cal-27 **(A)** and SAS **(B)** cells were treated with PMA (30 nM), TNF-α (10 ng/ml) or UVB (50 mJ/cm^2^) for 6 h, and immunofluorescence staining was performed to detect Blimp-1. Nuclei were counterstained with DAPI.

## Abbreviations

Blimp-1, B lymphocyte-induced maturation protein-1; EGF, epidermal growth factor; EGFR, EGF receptor; PMA, phorbol 12-myristate 13-acetate; Q-PCR, quantitative polymerase chain reaction; RIPA, radioimmunoprecipitation assay; ROS, reactive oxygen species; SCC, squamous cell carcinoma; TNF-α, tumor necrotic factor-α.
