# Supplementary figures and images for: Blimp-1 Upregulation by Multiple Ligands via EGFR Transactivation Inhibits Cell Migration in Keratinocytes and Squamous Cell Carcinoma
Source: Front Pharmacol. 2022 Feb 2;13:763678. doi: 10.3389/fphar.2022.763678 (PMC8847214; doi:10.3389/fphar.2022.763678)

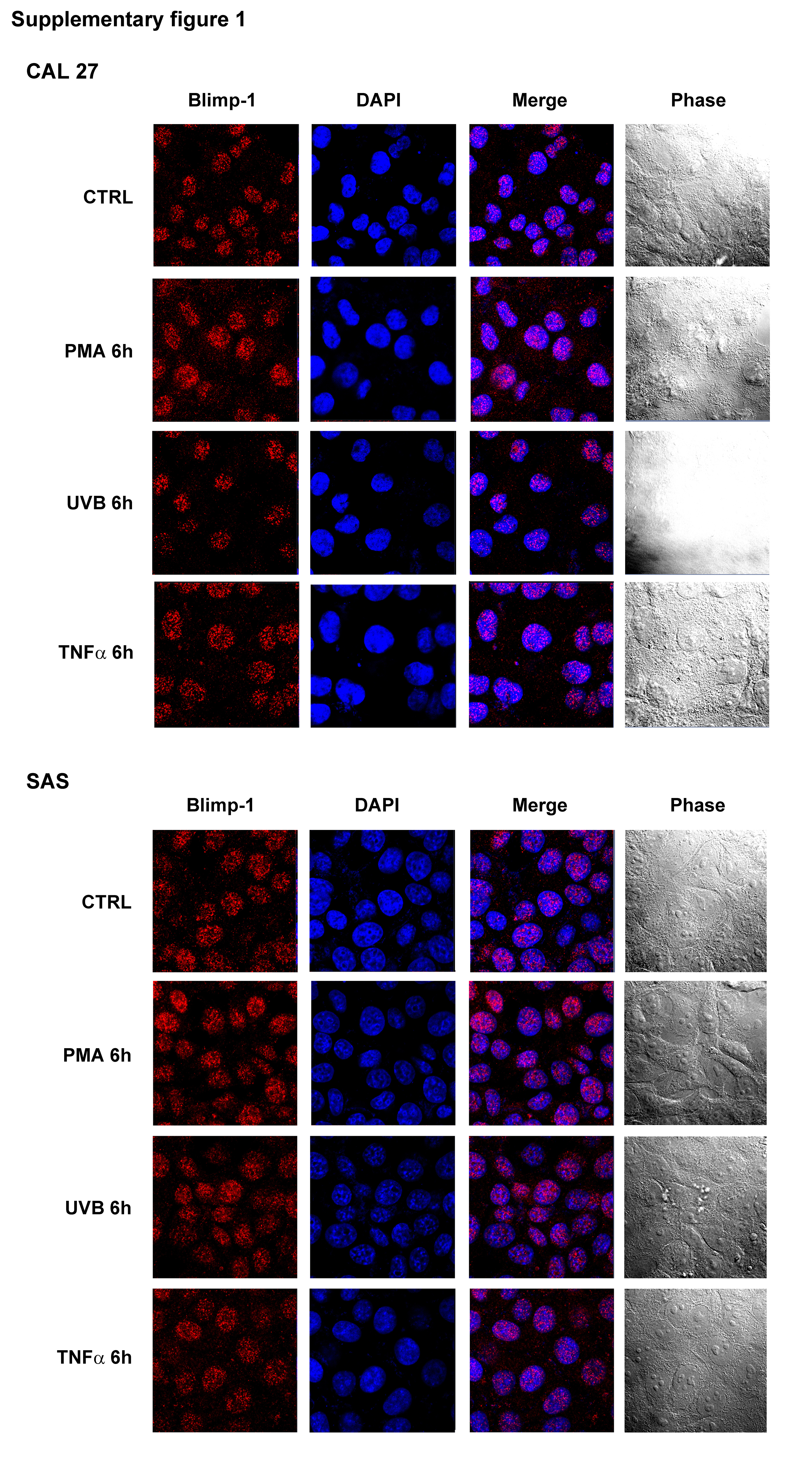

Supplement: Supplementary file 1 [file Image1.JPEG]
